# Infrared dermal thermometry is highly reliable in the assessment of patients with Charcot neuroarthropathy

**DOI:** 10.1186/s13047-020-00421-z

**Published:** 2020-09-14

**Authors:** Sarah M. Dallimore, Nicholas Puli, Daniel Kim, Michelle R. Kaminski

**Affiliations:** 1 Department of Podiatry, Eastern Health, Melbourne, Victoria 3128 Australia; 2 Department of Podiatry, Alfred Health, Melbourne, Victoria 3004 Australia; 3 Department of Quality Planning and Innovation, Eastern Health, Melbourne, Victoria 3128 Australia; 4 Discipline of Podiatry, School of Allied Health, Human Services and Sport, La Trobe University, Melbourne, Victoria 3086 Australia; 5 Department of Podiatry, St Vincent’s Hospital Melbourne, Melbourne, Victoria 3065 Australia

**Keywords:** Charcot foot, Diabetic foot, Neurogenic Arthropathy, Skin temperature, Thermometry

## Abstract

**Background:**

Charcot neuroarthropathy (Charcot foot) is a serious limb-threatening complication most commonly seen in individuals with diabetic peripheral neuropathy. Although dermal thermometry is widely used by clinicians to assist in the diagnosis, monitoring, and management of the disease, there is limited high-quality evidence to support its reliability. Therefore, this study investigated the intra-rater and inter-rater reliability of infrared dermal thermometry in patients with Charcot neuroarthropathy.

**Methods:**

We collected clinical, demographic, health status, and foot examination information on 32 adults with Charcot neuroarthropathy from a metropolitan high-risk foot service in Melbourne, Australia. Infrared dermal thermometry assessments were conducted by two independent raters at 10 anatomical sites of the Charcot foot using both a (i) touch and (ii) non-touch technique. Intra-rater and inter-rater reliability of the two assessment techniques were evaluated using intra-class correlation coefficients (ICCs), limits of agreement, standard error of measurement, and minimal detectable change statistics.

**Results:**

Mean age was 59.9 (standard deviation [SD], 10.5) years, 68.8% were male, average duration of diabetes was 20.6 (SD, 15.1) years, 71.9% had type 2 diabetes, 93.8% had peripheral neuropathy, 43.8% had peripheral arterial disease, and 50% had previous foot ulceration. Charcot foot most commonly affected the tarsometatarsal joints (38.9%), had a median duration of 2.8 (interquartile range [IQR], 1.3 to 5.9) months, and a large proportion were being treated with total contact casting (69.4%). Overall, there was good to excellent intra-rater and inter-rater relative reliability for the ‘touch’ technique (ICC, 0.87 to 0.99; ICC, 0.83 to 0.98, respectively), and excellent intra-rater and inter-rater relative reliability for the ‘non-touch’ technique (ICC, 0.93 to 0.99; ICC, 0.91 to 0.99, respectively). In addition, measurement error was found to be relatively low across the 10 anatomical sites.

**Conclusions:**

Infrared dermal thermometry can now be used with confidence in clinical and research settings to provide a reliable assessment of skin temperature in patients with Charcot neuroarthropathy, using either a touch or non-touch technique at 10 commonly used testing sites. A non-touch technique, however, was observed to have slightly higher reliability indicating it may be associated with less measurement error than the touch technique.

## Background

Charcot neuroarthropathy (CN), commonly referred to as ‘Charcot foot’, is a serious limb-threatening complication seen in individuals with peripheral neuropathy. Although diabetic neuropathy is the most common cause [1, 2], it can also result from other conditions with neuropathic manifestations, such as alcoholism and renal failure [1, 3]. The estimated prevalence of CN ranges from 0.08% in the general diabetes population to 13% in the diabetes high-risk foot population [1, 4].

CN is a progressive and destructive bone and joint disease that can affect single or multiple joints of the foot and ankle [1, 2, 5]. It is characterised by acute fractures, joint subluxation, dislocation and instability, and bony destruction [2, 6, 7]. Acute CN typically presents as a warm, erythematous, and oedematous foot [5]. Misdiagnosis in its early stages can lead to gross foot deformity, ulceration and amputation [7, 8]. Therefore, early detection and management are paramount.

Hand-held infrared dermal thermometry is a non-invasive skin temperature assessment used to assist clinical diagnosis, monitor disease progression and resolution, and guide management principles [7, 9–11]. A temperature difference of more than two degrees Celsius between the affected and non-affected Charcot foot is used as a clinical marker to detect acute (i.e. active) CN, while temperature differences of less than two degrees Celsius support safe withdrawal of immobilisation (e.g. total contact cast) [7, 10, 12, 13].

Commercially available, low-cost, hand-held infrared thermometers have been shown to have good accuracy, reliability and performance in assessing temperatures within a laboratory setting [14]. Other studies have shown a comparative level of agreement and repeatability between infrared thermometers and thermistor-type thermometers [15, 16]. Despite the wide clinical use of infrared dermal thermometry in the diagnosis, monitoring, and management of CN, there is surprisingly a lack of high-quality evidence to support its reliability. In addition, there is no consensus as to which anatomical sites should be included when performing this assessment. Common testing sites reported in the literature include: the hallux, medial 1st metatarsal head, lateral 5th metatarsal head, plantar metatarsal heads 1, 3 and 5, dorsal midfoot, 1st metatarsocuneiform joint, talonavicular joint, cuboid, plantar heel, and ankle [7, 11, 17, 18]. However, there have been no studies to date that have objectively compared the reliability of these testing sites.

Given the limitations of current evidence, this study aimed to investigate: (i) intra-rater and inter-rater reliability of infrared dermal thermometry using a ‘touch’ and ‘non-touch’ technique, and (ii) provide recommendations on the most reliable anatomical sites to test in patients with CN.

## Methods

### Ethics approval

This study was approved by the relevant institutional ethics committee (LR74–2014) and all participants gave written informed consent prior to enrolment and data collection.

### Participants

**Fig. 1:**
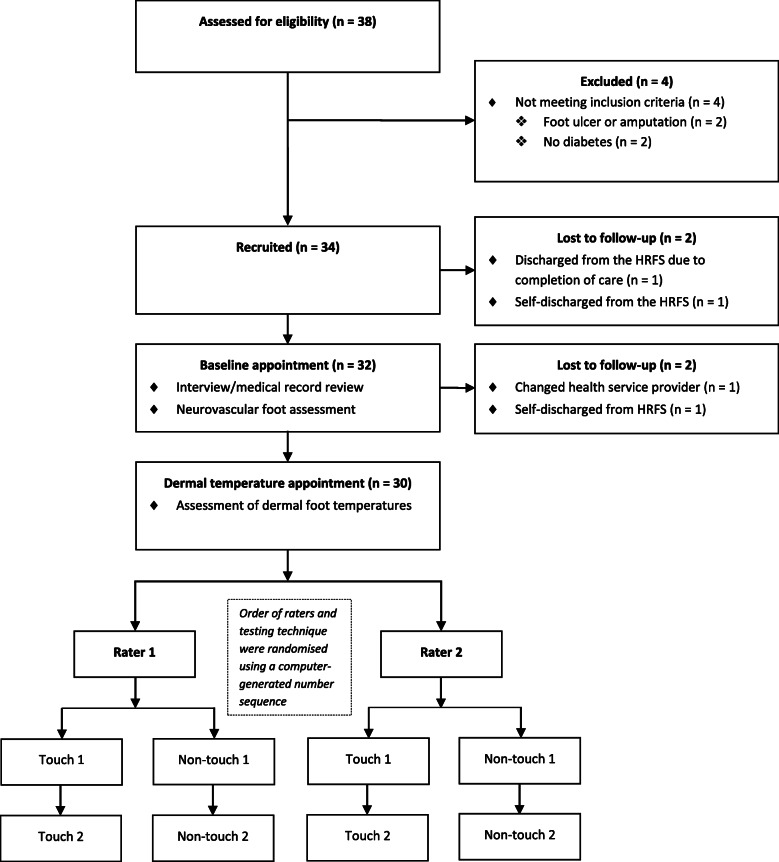
Diagram of study design and participant flow

Thirty-four participants with CN were consecutively recruited from a metropolitan high-risk foot service (HRFS) in Melbourne, Australia from February 2015 to December 2018 (Fig. 1 and Table 1). Eligibility for the study was determined by an interview and a non-invasive foot assessment. Participants were eligible if they had diabetes mellitus (type 1 or type 2), Charcot foot (defined as modified Eichenholtz stages 0 to 3) [19–21], were at least 18 years of age and were cognitively aware (i.e. they could provide informed consent). Participants were excluded if they had insufficient English skills to provide informed consent or follow instructions, had a current foot ulcer or had a lower extremity amputation (defined as a ‘complete loss of any part of the lower extremity’ [22], including any digit and/or partial foot amputation) on the Charcot foot.

**Table 1:** Participant characteristics

	Total (***N*** = 32)
Age, mean (SD), *years*	59.9 (10.5)
Male sex, n (%)	22 (68.8)
BMI, mean (SD), *kg/m*^*2*^	33.1 (7.9)
Diabetes mellitus	
Type 1, n (%)	9 (28.1)
Type 2, n (%)	23 (71.9)
HbA1c, mean (SD), *%*^a^	7.7 (1.3)
Duration, mean (SD), *years*	20.6 (15.1)
Previous Charcot foot (i.e. resolved), n (%)	8 (25.0)
Previous foot ulceration, n (%)	16 (50.0)
Dyslipidaemia, n (%)	20 (62.5)
Hypertension, n (%)	26 (81.3)
Ischaemic heart disease, n (%)	5 (15.6)
Congestive heart failure, n (%)	2 (6.3)
Cerebrovascular disease, n (%)	1 (3.1)
Osteoarthritis, n (%)	9 (28.1)
Inflammatory arthritis, n (%)	2 (6.3)

Data are n (%), unless otherwise specified. Percentages may not add up to 100%, as they are rounded to the nearest percent

*SD* Standard deviation, *BMI* Body mass index, *HbA1c* Glycated haemoglobin

^a^Maximum missing data were for HbA1c involving 2 participants overall (6.3%)

Two participants who were eligible and provided consent were lost to follow-up prior to data collection (one was discharged from the HRFS due to completion of care, and the other self-discharged from the HRFS). Thirty-two participants attended their baseline assessment (interview and neurovascular foot examination), but only 30 had their dermal temperatures measured (one self-discharged from the HRFS and one transferred to a different health service) (Fig. 1).

### Raters

Two independent raters were used in this study. Rater 1 (N.P.) was a podiatrist with 5 years’ clinical experience, and rater 2 (S.M.D.) was a senior podiatrist with 9 years’ clinical experience of using infrared dermal thermometry in patients with CN.

### Procedure

Written informed consent was obtained prior to the collection of any data. Data were collected over one to two appointments, which included a baseline assessment (interview and neurovascular foot examination) and the assessment of dermal temperatures. Figure 1 outlines the study design and flow of participants.

#### Baseline assessment

Data collection at baseline consisted of an interview with the participant, reviewing medical records, and conducting a non-invasive neurovascular foot assessment. Three examiners (S.M.D., D.K. and N.P.) were involved in the collection of baseline data and performed the foot assessments. Baseline variables relating to participant characteristics, comorbidities, and foot history can be found in Tables 1 and 2. Medical records were referred to for confirmation of medical and foot history.

**Table 2:** Foot history and neurovascular assessment

	Total (***N*** = 32)
Charcot neuroarthropathy, n (%)	
Right foot	12 (37.5)
Left foot	16 (50.0)
Bilateral	4 (12.5)
Total number of Charcot feet	36
Charcot foot pattern, no. (%)^a^	
Forefoot	2 (5.6)
Tarsometatarsal joints	14 (38.9)
NC, TN, CC joints	10 (27.8)
Ankle and subtalar joints	1 (2.8)
Calcaneus	0 (0)
Mixed	9 (25.0)
Duration of Charcot foot, median (IQR), *months*^*b*^	2.8 (1.3 to 5.9)
Current Charcot treatment, no. (%)^a^	
Total contact cast	25 (69.4)
CAM walker	5 (13.9)
Charcot restraint orthotic walker	1 (2.8)
Orthopaedic footwear and orthoses	3 (8.3)
Other	2 (5.6)
Previous Charcot foot (i.e. resolved), n (%)	8 (25.0)
Right foot, no. (%)	6 (18.8)
Charcot foot pattern, no. (%)	
Forefoot	0 (0)
Tarsometatarsal joints	4 (66.7)
NC, TN, CC joints	2 (33.3)
Ankle and subtalar joints	0 (0)
Calcaneus	0 (0)
Mixed	0 (0)
Left foot, no. (%)	2 (6.3)
Charcot foot pattern, no. (%)	
Forefoot	0 (0)
Tarsometatarsal joints	1 (50.0)
NC, TN, CC joints	0 (0)
Ankle and subtalar joints	1 (50.0)
Calcaneus	0 (0)
Mixed	0 (0)
Previous foot ulceration, n (%)	16 (50.0)
Mean (SD), range^c^	1 (1.2), 1–4
Median (IQR)^c^	1 (1 to 2)
Total number of previous foot ulcers	32
Right foot, total no. (%)	21 (65.6)
Location, total no. (%)	
Digits	11 (52.4)
Plantar forefoot	6 (28.6)
Plantar midfoot	1 (4.8)
Dorsal foot	2 (9.5)
Heel	0 (0)
Multiple areas of the foot	1 (4.8)
Duration, mean (SD), *months*	6.8 (5.3)
Left foot, total no. (%)	11 (34.4)
Location, total no. (%)	
Digits	5 (45.5)
Plantar forefoot	1 (9.1)
Plantar midfoot	4 (36.4)
Dorsal foot	0 (0)
Heel	0 (0)
Multiple areas of the foot	1 (9.1)
Duration, mean (SD), *months*	4.1 (3.5)
Peripheral neuropathy, n (%)^d,e^	30 (93.8)
Vibration perception threshold, mean (SD), *volts*^*f*^	
Right foot	32.4 (12.5)
Left foot	27.6 (13.0)
Protective sensation, median (IQR), *(/3 sites)*	
Right foot	0 (0 to 1)
Left foot	0 (0 to 2)
Peripheral arterial disease, n (%)^g^	14 (43.8)
Ankle-brachial pressure index, mean (SD)^f^	
Right foot	1.28 (0.24)
Left foot	1.28 (0.28)
Toe-brachial pressure index, mean (SD)^f^	
Right foot	1.00 (0.30)
Left foot	0.99 (0.41)
Arterial calcification, n (%)^h^	12 (37.5)

Data are n (%), unless otherwise specified. Percentages may not add up to 100%, as they are rounded to the nearest percent

*NC* Naviculocuneiform, *TN* Talonavicular, *CC* Calcaneocuboid, *IQR* Interquartile range, *CAM* Controlled ankle motion, *SD* Standard deviation

^a^Calculated from total number of Charcot feet (i.e. 36 Charcot feet)

^b^Calculated from Charcot foot included in the dermal temperature data analysis (i.e. for those that had bilateral Charcot, only one foot was included in the analysis)

^c^Previous foot ulcers per participant

^d^Peripheral neuropathy was defined as vibration perception threshold > 25 V (either foot); and/or monofilament score < 3/3 (either foot)

^e^If an amputation was present on a non-Charcot foot (e.g. hallux), an appropriate alternative site was chosen to complete the neurological test (e.g. styloid process)

^f^Maximum missing data were for vibration perception threshold involving 11 participants overall (34.4%). Missing data were for ankle-brachial pressure index (right, *n* = 7; left, *n* = 5), toe-brachial pressure index (right, *n =* 1; left, *n* = 4), and vibration perception threshold (right and left, *n* = 11)

^g^Peripheral arterial disease was defined as absence of ≥2 pedal pulses; ankle-brachial pressure index ≤0.9 (either leg/foot); and/or toe-brachial pressure index ≤0.6 (either foot)

^h^Arterial calcification was defined as an ankle-brachial pressure index > 1.3 or non-compressible peripheral arteries (i.e. ankle systolic reading of > 240 mmHg)

Participants had their height and weight measured to determine their body mass index. The neurovascular foot assessments were performed as per a previous protocol [23]. Neurological status was determined by evaluating the vibration perception threshold (VPT) with a Horwell® neurothesiometer at the apex of the hallux (average of three measurements), and protective sensation with a Bailey Instruments Ltd.® Semmes-Weinstein 5.07/10 g monofilament at the plantar hallux and plantar 1st and 5th metatarsophalangeal joints. Peripheral neuropathy was defined as VPT > 25 V and/or monofilament score < 3/3 sites in either foot. Arterial status was determined by evaluating pedal pulses (dorsalis pedis and posterior tibial), ankle brachial pressure indices (ABPI) using a Hadeco ES100V3 Bidop® Doppler ultrasound and a WelchAllyn® sphygmomanometer and cuff, and toe-brachial pressure indices (TBPI) using a Hadeco® photoplethysmography probe and toe cuff. Peripheral arterial disease was defined as absence of ≥2 pedal pulses, ABPI ≤0.9, and/or TBPI ≤0.6 [23].

#### Dermal temperature assessment

Two independent raters (N.P. and S.M.D.) were involved in the collection of dermal temperature data, which was performed during the participants’ usual review appointments in the HRFS. In cases of bilateral CN, the most acute Charcot foot at the time of data collection was included in the statistical analysis of the temperature data.

**Fig. 2:**
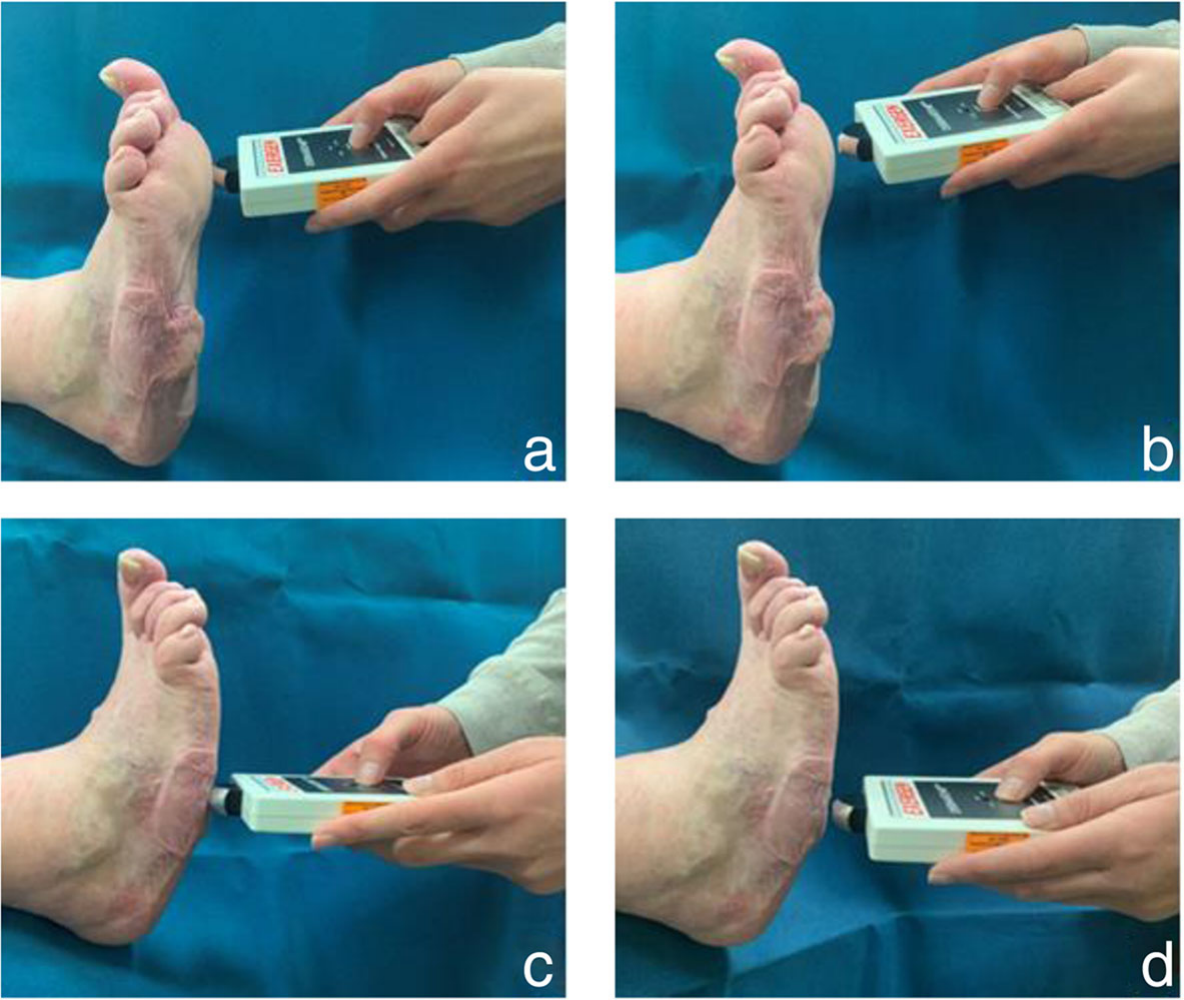
Touch and non-touch techniques. (**a**) Touch technique at site 1. (**b**) Non-touch technique at site 1. (**c**) Touch technique at site 4. (**d**) Non-touch technique at site 4. Participant photographs reprinted with permission

Room and outside temperatures were recorded prior to data collection. The infrared dermal thermometer ‘DermaTemp (DT)-1001’ from Exergen Corporation® (Watertown, Massachusetts) was chosen for this study, as it is a commonly used device in clinical practice [6, 11, 12, 24, 25]. Features of this device include: 1:1 distance-to-spot ratio, sensor diameter of 3 mm, fixed emissivity, and is accurate to ±0.1 °C [7, 14]. To ensure stabilisation of skin temperature prior to assessment, a minimum 15-min acclimatisation period was used (i.e. time from removal of footwear/hosiery) [26]. Dermal temperatures were assessed by the two independent raters on the Charcot foot using a (i) touch and (ii) non-touch technique at 10 anatomical sites of interest (Table 3). The ‘touch’ technique was defined as lightly pressing the device against the skin at the point of interest during measurement. The ‘non-touch’ technique was defined as holding the device approximately 5 mm away from the skin surface at the point of interest during measurement (Fig. 2) [11, 27]. A randomised computer-generated number sequence was used to determine the order of raters and technique used (i.e. touch versus non-touch).

**Table 3:** Anatomical testing sites

1	Plantar 1st metatarsal head
2	Plantar 3rd metatarsal head
3	Plantar 5th metatarsal head
4	Plantar aspect of the base of the 5th metatarsal (styloid process)
5	Dorsal aspect of the base of the 3rd metatarsal
6	Medial aspect of the base of the 1st metatarsal
7	Medial aspect of the navicular
8	Plantar medial tubercle of the calcaneus
9	Medial malleolus
10	Lateral malleolus

Following each session of the dermal temperature assessments, data sheets were sealed in opaque envelopes. Therefore, raters were blinded to each other’s results and were not able to revise their own results between tests. Independent members of the study team (D.K. and M.R.K.) entered the data into a spreadsheet on the two raters’ behalf.

### Statistical analysis

Participant characteristics were calculated and expressed as mean (standard deviation, SD), median (interquartile range, IQR), or number (proportion). Relative reliability was evaluated using intra-class correlation coefficients (ICCs) with corresponding 95% confidence intervals (CI) based on a single rating, absolute agreement, two-way random-effects model. ICC values greater than 0.9 were considered excellent, 0.75 to 0.9 considered good, 0.5 to 0.75 considered moderate, and less than 0.5 considered poor [28]. Absolute reliability was evaluated using standard error of measurement (SEM), minimal detectable change (MDC) and limits of agreement (LOA) statistics. IBM SPSS version 23.0 (IBM Corp, Somers, NY, USA) was used for statistical analysis.

## Results

We collected data on 32 adults with diabetes mellitus and CN. The mean age was 59.9 (SD, 10.5) years, 68.8% were male, and the average body mass index was 33.1 (SD, 7.9) kg/m^2^. The majority had type 2 diabetes (71.9%). The mean duration of diabetes was 20.6 (SD, 15.1) years and the average glycated haemoglobin was 7.7% (SD, 1.3). Peripheral neuropathy, peripheral arterial disease and previous foot ulceration were highly prevalent (93.8, 43.8 and 50%, respectively). Overall, there were a total of 36 Charcot feet among the 32 participants (four participants had bilateral CN). Charcot foot most commonly affected the tarsometatarsal joints (38.9%), followed by the naviculocuneiform, talonavicular, and calcaneocuboid joints (27.8%). The median duration of Charcot foot at the time of the baseline assessment was 2.8 (IQR, 1.3 to 5.9) months. A large proportion of Charcot feet were receiving total contact casting treatment (69.4%). A quarter of the sample (*n* = 8) had a previous (i.e. resolved) Charcot foot. Participant characteristics, foot history, and foot examination information are shown in Tables 1 and 2.

The average room and outside temperatures recorded at the first dermal temperature assessment were 22.4 °C (SD, 1.0) and 17.3 °C (SD, 5.4), respectively. Following the minimum 15-min acclimatisation period, the median time to performing the dermal temperature assessments was 1.1 (IQR, 0.3 to 5.0) minutes. The results of the relative and absolute reliability of the dermal temperature assessments in the units of measurement degrees Celsius (°C) are shown in Tables 4 and 5.

**Table 4:** Intra-rater reliability for the touch and non-touch technique

	Site	Temperature test (°C) Mean (SD)		Temperature retest (°C) Mean (SD)		ICC	95% CI		MD	SDd	LOA		SEM	MDC
***Touch technique***														
***Rater 1***	1	28.00	(1.63)	28.12	(1.78)	0.98	0.96	0.99	0.11	0.32	−0.55	0.78	0.24	0.67
	2	28.51	(1.50)	28.56	(1.58)	0.98	0.97	0.99	0.05	0.28	−0.52	0.62	0.20	0.54
	3	27.87	(1.46)	27.94	(1.54)	0.97	0.93	0.98	0.06	0.38	−0.72	0.85	0.27	0.75
	4	28.13	(1.32)	28.29	(1.41)	0.95	0.89	0.98	0.16	0.42	−0.70	1.02	0.31	0.86
	5	29.72	(1.06)	29.75	(1.05)	0.94	0.88	0.97	0.03	0.36	−0.70	0.77	0.25	0.70
	6	28.92	(1.23)	29.05	(1.31)	0.95	0.90	0.98	0.13	0.37	−0.63	0.89	0.27	0.76
	7	29.48	(1.17)	29.61	(1.25)	0.93	0.85	0.96	0.13	0.46	−0.81	1.06	0.33	0.91
	8	27.41	(1.79)	27.55	(1.74)	0.89	0.79	0.95	0.14	0.82	−1.55	1.83	0.58	1.61
	9	29.74	(1.18)	29.75	(1.20)	0.98	0.96	0.99	0.01	0.25	−0.49	0.51	0.17	0.48
	10	29.47	(1.42)	29.52	(1.46)	0.97	0.94	0.99	0.04	0.36	−0.69	0.78	0.25	0.70
***Rater 2***	1	28.05	(1.65)	28.11	(1.70)	0.98	0.96	0.99	0.06	0.31	−0.58	1.50	0.22	0.61
	2	28.58	(1.48)	28.61	(1.60)	0.98	0.96	0.99	0.03	0.31	−0.61	1.56	0.22	0.60
	3	27.98	(1.47)	27.96	(1.56)	0.99	0.97	0.99	−0.02	0.25	−0.54	1.35	0.18	0.50
	4	28.19	(1.29)	28.26	(1.37)	0.98	0.95	0.99	0.07	0.30	−0.54	1.41	0.21	0.58
	5	29.65	(1.02)	29.81	(1.03)	0.87	0.74	0.93	0.16	0.51	−0.89	2.33	0.37	1.04
	6	29.04	(1.15)	29.04	(1.29)	0.91	0.82	0.96	0.00	0.52	−1.06	2.70	0.36	1.01
	7	29.41	(1.11)	29.57	(1.14)	0.93	0.85	0.97	0.16	0.40	−0.66	1.76	0.30	0.83
	8	27.57	(1.65)	27.63	(1.69)	0.98	0.95	0.99	0.05	0.37	−0.71	1.82	0.26	0.72
	9	29.71	(1.13)	29.73	(1.12)	0.97	0.93	0.98	0.02	0.30	−0.60	1.53	0.21	0.58
	10	29.47	(1.38)	29.62	(1.37)	0.96	0.91	0.98	0.15	0.39	−0.65	1.71	0.29	0.80
***Non-touch technique***														
***Rater 1***	1	28.07	(1.69)	28.07	(1.70)	0.99	0.98	1.00	0.00	0.23	−0.47	1.19	0.16	0.45
	2	28.55	(1.51)	28.49	(1.63)	0.98	0.97	0.99	−0.06	0.29	−0.65	1.61	0.20	0.57
	3	27.99	(1.49)	27.91	(1.58)	0.99	0.97	0.99	−0.08	0.25	−0.59	1.46	0.18	0.50
	4	28.23	(1.30)	28.16	(1.40)	0.93	0.87	0.97	−0.07	0.50	−1.08	2.71	0.35	0.96
	5	29.73	(1.11)	29.75	(1.11)	0.96	0.92	0.98	0.02	0.31	−0.61	1.57	0.22	0.60
	6	28.93	(1.29)	29.08	(1.23)	0.93	0.86	0.97	0.16	0.44	−0.75	1.97	0.33	0.90
	7	29.51	(1.18)	29.54	(1.22)	0.93	0.86	0.97	0.03	0.45	−0.90	2.28	0.32	0.87
	8	27.47	(1.61)	27.48	(1.66)	0.97	0.93	0.98	0.01	0.44	−0.89	2.26	0.31	0.85
	9	29.74	(1.27)	29.83	(1.22)	0.97	0.93	0.98	0.09	0.31	−0.55	1.44	0.23	0.63
	10	29.55	(1.37)	29.60	(1.44)	0.98	0.95	0.99	0.06	0.31	−0.57	1.48	0.22	0.60
***Rater 2***	1	28.04	(1.68)	28.16	(1.66)	0.99	0.97	1.00	0.11	0.19	−0.28	0.76	0.16	0.44
	2	28.55	(1.55)	28.59	(1.54)	0.99	0.98	1.00	0.04	0.23	−0.42	1.09	0.16	0.45
	3	27.92	(1.51)	27.98	(1.53)	0.99	0.97	0.99	0.06	0.25	−0.45	1.16	0.18	0.50
	4	28.23	(1.38)	28.28	(1.42)	0.98	0.97	0.99	0.05	0.25	−0.46	1.19	0.18	0.49
	5	29.83	(1.08)	29.87	(1.03)	0.97	0.94	0.99	0.05	0.26	−0.48	1.24	0.18	0.51
	6	29.16	(1.23)	29.14	(1.36)	0.97	0.94	0.99	−0.02	0.33	−0.69	1.75	0.23	0.63
	7	29.48	(1.17)	29.59	(1.16)	0.97	0.93	0.99	0.12	0.28	−0.46	1.22	0.21	0.59
	8	27.66	(1.74)	27.66	(1.69)	0.99	0.97	0.99	0.00	0.29	−0.60	1.52	0.20	0.56
	9	29.71	(1.18)	29.88	(1.13)	0.96	0.89	0.98	0.17	0.29	−0.43	1.16	0.23	0.65
	10	29.58	(1.44)	29.64	(1.48)	0.99	0.98	1.00	0.06	0.20	−0.35	0.91	0.15	0.40

Units of measurement are degrees Celsius (°C)

*SD* Standard deviation, *ICC* Intra-class correlation coefficient, *CI* Confidence interval, *MD* Mean difference, *SDd* Standard deviation difference, *LOA* Limits of agreement, *SEM* Standard error of measurement, *MDC* Minimal detectable change

**Table 5:** Inter-rater reliability for the touch and non-touch technique

	Site	Temperature test (°C) Mean (SD)		Temperature retest (°C) Mean (SD)		ICC	95% CI		MD	SDd	LOA		SEM	MDC
***Touch technique***														
	1	28.00	(1.63)	28.05	(1.65)	0.98	0.96	0.99	0.05	0.34	−0.65	1.66	0.24	0.66
	2	28.51	(1.50)	28.58	(1.48)	0.98	0.95	0.99	0.07	0.33	−0.60	1.55	0.23	0.64
	3	27.87	(1.46)	27.98	(1.47)	0.92	0.85	0.96	0.11	0.58	−1.07	2.76	0.41	1.13
	4	28.13	(1.32)	28.19	(1.29)	0.93	0.85	0.96	0.06	0.50	−0.97	2.49	0.35	0.98
	5	29.72	(1.06)	29.65	(1.02)	0.92	0.84	0.96	−0.07	0.42	−0.93	2.34	0.30	0.83
	6	28.92	(1.23)	29.04	(1.15)	0.90	0.81	0.95	0.11	0.52	−0.96	2.49	0.37	1.03
	7	29.48	(1.17)	29.41	(1.11)	0.83	0.68	0.92	−0.07	0.67	−1.44	3.61	0.47	1.30
	8	27.41	(1.79)	27.57	(1.65)	0.96	0.91	0.98	0.16	0.48	−0.82	2.16	0.35	0.98
	9	29.74	(1.18)	29.71	(1.13)	0.89	0.79	0.95	−0.03	0.54	−1.14	2.87	0.38	1.05
	10	29.47	(1.42)	29.47	(1.38)	0.95	0.89	0.97	0.00	0.47	−0.96	2.42	0.33	0.90
***Non-touch technique***														
	1	28.07	(1.69)	28.55	(1.68)	0.99	0.99	1.00	−0.03	0.20	−0.44	1.10	0.14	0.39
	2	28.55	(1.51)	28.04	(1.55)	0.99	0.97	0.99	0.00	0.27	−0.55	1.40	0.19	0.52
	3	27.99	(1.49)	27.92	(1.51)	0.97	0.93	0.98	−0.07	0.40	−0.88	2.20	0.28	0.78
	4	28.23	(1.30)	28.23	(1.38)	0.94	0.88	0.97	0.00	0.47	−0.96	2.43	0.33	0.90
	5	29.73	(1.11)	29.83	(1.08)	0.92	0.85	0.96	0.10	0.42	−0.77	2.00	0.30	0.84
	6	28.93	(1.29)	29.16	(1.23)	0.92	0.81	0.96	0.23	0.46	−0.71	1.92	0.36	0.99
	7	29.51	(1.18)	29.48	(1.17)	0.91	0.82	0.96	−0.03	0.51	−1.07	2.69	0.35	0.98
	8	27.47	(1.61)	27.66	(1.74)	0.93	0.86	0.97	0.19	0.61	−1.05	2.76	0.44	1.23
	9	29.74	(1.27)	29.71	(1.18)	0.92	0.83	0.96	−0.03	0.51	−1.07	2.70	0.35	0.98
	10	29.55	(1.37)	29.58	(1.44)	0.98	0.95	0.99	0.03	0.31	−0.60	1.53	0.21	0.59

Units of measurement are degrees Celsius (°C)

*SD* Standard deviation, *ICC* Intra-class correlation coefficient, *CI* Confidence interval, *MD* Mean difference, *SDd* Standard deviation difference, *LOA* Limits of agreement, *SEM* Standard error of measurement, *MDC* Minimal detectable change

### Intra-rater reliability

Relative reliability was found to be ‘good to excellent’ for the touch technique and ‘excellent’ for the non-touch technique across the 10 sites. ICCs ranged from 0.87 to 0.99 for the touch technique, and 0.93 to 0.99 for the non-touch technique across the two raters. Measurement error was found to be relatively low across the 10 sites for the two raters. MDC values ranged from 0.48 to 1.61 °C (SEM, 0.17 to 0.58 °C) for the touch technique, and 0.40 to 0.96 °C (SEM, 0.15 to 0.35 °C) for the non-touch technique (Table 4).

### Inter-rater reliability

Relative reliability was found to be ‘good to excellent’ for the touch technique and ‘excellent’ for the non-touch technique across the 10 sites. ICCs ranged from 0.83 to 0.98 for the touch technique, and 0.91 to 0.99 for the non-touch technique across the two raters. Between the two raters, measurement error was found to be relatively low across the 10 sites. MDC values ranged from 0.64 to 1.30 °C (SEM, 0.23 to 0.47 °C) for the touch technique, and 0.39 to 1.23 °C (SEM, 0.14 to 0.44 °C) for the non-touch technique (Table 5).

## Discussion

This study found that infrared dermal thermometry is a highly reliable tool in the clinical assessment of patients with CN. Overall, there was ‘good to excellent’ intra-rater and inter-rater reliability for the touch technique, and ‘excellent’ intra-rater and inter-rater reliability for the non-touch technique. In addition, measurement error was relatively low across the 10 anatomical sites tested. These findings suggest that either a touch or non-touch technique can be used confidently in clinical practice or research settings.

Intra-rater reliability was found to be better than inter-rater reliability for both techniques. Given that infrared thermometry is used to compare dermal temperatures between an affected and non-affected Charcot foot at a particular point in time (rather than across different days), and that the full assessment is most commonly performed by one clinician at any one point in time, intra-rater reliability is most relevant to clinical practice when performing this assessment. That being said, this study also showed high inter-rater reliability for both techniques, therefore, clinicians can remain confident with the temperature readings if the assessment is shared between clinicians.

Interestingly, a non-touch technique was observed to have slightly higher reliability and lower measurement error than the touch technique. This finding was unexpected as the non-touch technique required examiners to estimate a 5 mm distance from the device to the skin surface. One possible explanation for this finding is that the amount of pressure being applied by the raters for the touch technique may have varied, and therefore caused some inconsistencies in the temperature readings. This finding is in contrast to recommendations outlined in the Exergen Corporation® DT-1001 user manual, which states for “maximum accuracy the probe must contact the surface at the point of interest” [29]. While a touch technique may improve accuracy (i.e. temperature measured is reflective of the true temperature), our findings suggest that a non-touch technique is slightly more reliable than the touch technique. The current study focused on investigating the reliability of dermal temperature assessments (i.e. test-retest performance), which from a clinical perspective, is considered most important in establishing temperature differences between an affected and non-affected Charcot foot.

Measurement error was found to be relatively low across the 10 testing sites for the two raters and techniques, as indicated by the MDC values obtained (Tables 3 and 4). The MDC value represents an estimate of the amount of change required (in this case to dermal temperatures) for the change to be considered ‘real’, which is over and above measurement error [30]. As an example, if the MDC value is equal to 0.5, a change in Charcot foot temperature that is ≤0.5 °C in a test-retest scenario (e.g. trial 1 = 28 °C then trial 2 = 27.5 °C) would be accountable to measurement error (e.g. operator error). Inversely, any change in temperature that is greater than 0.5 °C would be due to a true change in temperature. Of the 10 anatomical sites assessed in this study, sites 1 and 2 (plantar 1st and 3rd metatarsal heads) were observed to have consistently high reliability across the raters and techniques. These sites were less likely to be affected by CN in this study, as a large proportion of participants presented with midfoot Charcot. Therefore, a potential explanation for this finding is that sites correlating to joints affected by CN may be less reliable and requires further investigation. Considering previous studies have found that sites of elevated dermal temperature correlate with the joints affected by CN [7, 11, 17], the presentation or pattern of CN is often variable with multiple joints affected, and the number of sites that should be tested in patients with CN is currently unclear, dermal temperature assessments should include an appropriate number and range of relevant sites to account for this. Given that there was no discernible difference in reliability across the 10 anatomical sites used in this study, and the assessment is non-invasive and quick to perform, the 10-site protocol presented in this study can be confidently used in clinical practice.

There are several potential limitations of this study. First, we did not assess different acclimatisation periods and whether they have an impact on the reliability of dermal temperature assessments. As per a previous study’s [26] recommendations, we used a minimum 15-min acclimatisation period to ensure stabilisation of foot temperatures prior to testing. Despite our best efforts to be consistent in the timing of dermal temperature assessments among participants, there was some variability from the end of the 15-min acclimatisation period to the start of the dermal temperature assessment. To adjust for this, room and outside temperatures were recorded prior to data collection to ensure no significant changes to foot temperatures may have occurred. Second, both raters in this study had high levels of clinical experience using infrared dermal thermometry to assess patients with CN. Therefore, it remains unclear whether less experienced clinicians or those with no experience would have reduced reliability when conducting this assessment. Third, we did not assess for the accuracy of the infrared dermal thermometer, therefore, our results can only be used for interpreting reliability of this assessment. Fourth, it remains unclear whether room or outside temperature changes may have had an impact on the dermal temperature measurements. However, as room and outside temperatures were recorded for each assessment by the two raters, and all participants were assessed in the same clinical environment, this was unlikely. In addition, temperature differences between an affected and non-affected Charcot foot is the key clinical factor when assessing CN, therefore, climate control of the environment is not as essential. Fifth, this study used either a touch or non-touch ‘pin-point’ testing method, therefore, our results are not generalisable to clinicians that use the ‘scanning’ method (i.e. moving probe around anatomical site to get highest reading). Sixth, our findings are only generalisable to patients with diabetes-related CN (i.e. not from renal disease, alcoholism etc.). Finally, recall bias may have been present (e.g. participants self-reported previous foot ulcers and CN duration), however medical records were referred to if clarification was needed, so this was unlikely.

There are several strengths of this study. To our knowledge, it is the first study to investigate intra-rater and inter-rater reliability of infrared dermal thermometry in CN. This study had a rigorous inclusion and exclusion criteria, sufficient sample size [28], robust study protocol, and our findings are able to be generalised to clinical practice as our study included patients with CN from a HRFS.

As this is the first study to evaluate the reliability of infrared dermal thermometry in the assessment of patients with CN, there is limited capacity to compare the current study findings to previous literature. Our finding that infrared dermal thermometry is a highly reliable assessment tool, is consistent with a previous laboratory-based study [14]. This study found high reliability (*r* > 0.989) between temperature change measurements of two raters when comparing nine commercially available infrared thermometers (including the Exergen Corporation® DT-1000) [14]. Infrared thermometry has also been shown to have high correlations between: (i) different thermometers (*r* > 0.80) in assessing other high-risk foot conditions such as peri-wound temperatures and (ii) the assessment of skin temperature via palpation (*r*_*s*_ = 0.81, *p* < 0.000) [27, 31].

Further research is needed to establish an evidence-based protocol for dermal temperature assessment in patients with CN. Future research may be directed towards investigating the reliability of: (i) a touch and/or non-touch technique of the ‘pin-point’ versus ‘scanning’ method, (ii) the comparison of an affected and non-affected Charcot foot, (iii) different acclimatisation periods, and (iv) the level of clinician experience and/or comparisons between different health professionals in performing dermal temperature assessments.

## Conclusions

This is the first study to assess the reliability of dermal temperature assessments in patients with CN. Infrared dermal thermometry can now be used with confidence in clinical and research settings to provide a reliable assessment of skin temperature in patients with CN, using either a touch or non-touch technique at 10 commonly used testing sites. A non-touch technique, however, was observed to have slightly higher reliability indicating it may be associated with less measurement error than the touch technique.

## Supplementary information

**Additional file 1: **Adobe professional (.pdf). Screening tool and data collection form. **Description of data:** Screening tool used for prospective participants and data collection form used to collate the baseline and dermal temperature data.

## Data Availability

The datasets used and/or analysed during the current study are available from the corresponding author on reasonable request.

## Abbreviations

ABPI: Ankle-brachial pressure index
CI: Confidence interval
CN: Charcot neuroarthropathy
DT: DermaTemp
HRFS: High-risk foot service
ICC: Intraclass correlation coefficient
IQR: Interquartile range
LOA: Limits of agreement
MDC: Minimal detectable change
SD: Standard deviation
SEM: Standard error of measurement
TBPI: Toe-brachial pressure index
VPT: Vibration perception threshold
