# Supplementary material for: Infrared dermal thermometry is highly reliable in the assessment of patients with Charcot neuroarthropathy
Source: J Foot Ankle Res. 2020 Sep 14;13:56. doi: 10.1186/s13047-020-00421-z (PMC7489208; doi:10.1186/s13047-020-00421-z)
Supplement: Supplementary file 1 — Additional file 1: Adobe professional (.pdf). Screening tool and data collection form. Description of data: Screening tool used for prospective participants and data collection form used to collate the baseline and dermal temperature data. [file 13047_2020_421_MOESM1_ESM.pdf]

Screening Tool / Data Collection Form

SCREENING FOR ELIGIBILITY

Date of assessment: ________

Diabetes mellitus:	Yes	☐	No	☐	*If NO, exclude*
Charcot foot:	Yes	☐	No	☐	*If NO, exclude*
≥18 years of age:	Yes	☐	No	☐	*If NO, exclude*
Cognitively aware:	Yes	☐	No	☐	*If NO, exclude*
Sufficient English skills:	Yes	☐	No	☐	*If NO, exclude*
Willing to give informed consent:	Yes	☐	No	☐	*If NO, exclude*
Current foot ulcer on Charcot foot:	Yes	☐	No	☐	*If YES, exclude*
Previous amputation on Charcot foot:	Yes	☐	No	☐	*If YES, exclude*

*** If participant satisfies all eligibility criteria, continue with assessment.**

BASELINE ASSESSMENT

Participant characteristics

Date of birth: / /

Sex: Male ☐1 Female ☐2

Height: ________m Weight: ________kg BMI (kg/m²): ________

Comorbidities

Diabetes mellitus
Type of diabetes: Type 1 ☐1 Type 2 ☐2

Date of diagnosis: ____________ Duration of diabetes (months): ____________

Latest HbA1c result: ____________ (%)

Dyslipidaemia Yes ☐1 No ☐2

Hypertension Yes ☐1 No ☐2

Ischaemic heart disease Yes ☐1 No ☐2

Congestive heart failure Yes ☐1 No ☐2

Cerebrovascular disease Yes ☐1 No ☐2

Osteoarthritis Yes ☐1 No ☐2

Inflammatory arthritis Yes ☐1 No ☐2

Other____________

<u>Foot history</u>

Previous foot ulcer

Left foot:	Yes	☐ *1*	No	☐ *2*
Right foot:	Yes	☐ *1*	No	☐ *2*

Location of previous foot ulcer

Left Foot:

☐ *1* Digits
☐ *2* Plantar forefoot
☐ *3* Plantar midfoot
☐ *4* Dorsal foot
☐ *5* Heel
☐ *6* Multiple areas of the foot

Right Foot:

☐ *1* Digits
☐ *2* Plantar forefoot
☐ *3* Plantar midfoot
☐ *4* Dorsal foot
☐ *5* Heel
☐ *6* Multiple areas of the foot

Duration of previous foot ulcer

Left foot: Duration (months): _____________

Right foot: Duration (months): _____________

<u>Charcot foot history</u>

Charcot neuroarthropathy

Left foot:

Date of diagnosis: _____________ Duration of Charcot foot (months): _____________

Charcot foot pattern:

☐ *1* Forefoot
☐ 2 Tarsometatarsal joints
☐ *3* Naviculocuneiform, talonavicular + calcaneocuboid joints
☐ *4* Ankle and subtalar joints
☐ *5* Calcaneus
☐ *6* Mixed _____________ (describe)

Current treatment:

☐1 Total contact cast
☐2 Cam Walker
☐3 Charcot Restraint Orthotic Walker (CROW)
☐4 Orthopaedic footwear and/or orthoses
☐5 Other

Duration of treatment (months): ____________

Right foot:

Date of diagnosis: ____________ Duration of Charcot foot (months): ____________
Charcot foot pattern:

☐1 Forefoot
☐2 Tarsometatarsal joints
☐3 Naviculocuneiform, talonavicular + calcaneocuboid joints
☐4 Ankle and subtalar joints
☐5 Calcaneus
☐6 Mixed ____________ (describe)

Current treatment:

☐1 Total contact cast
☐2 Cam Walker
☐3 Charcot Restraint Orthotic Walker (CROW)
☐4 Orthopaedic footwear and/or orthoses
☐5 Other

Duration of treatment (months): ____________

Previous Charcot foot (i.e. resolved)

Left foot:

Charcot foot pattern:

☐1 Forefoot
☐2 Tarsometatarsal joints
☐3 Naviculocuneiform, talonavicular + calcaneocuboid joints
☐4 Ankle and subtalar joints
☐5 Calcaneus
☐6 Mixed ____________ (describe)

Right foot:

Charcot foot pattern:

☐1 Forefoot

☐2 Tarsometatarsal joints
☐3 Naviculocuneiform, talonavicular + calcaneocuboid joints
☐4 Ankle and subtalar joints
☐5 Calcaneus
☐6 Mixed ____________ (describe)

Neurological assessment

Vibration perception threshold (Neurothesiometer)

	Trial 1 (V)	Trial 2 (V)	Trial 3 (V)	Average (V)
**Left foot**				
**Right foot**				

Note. Vibration perception threshold >25 V will indicate peripheral neuropathy.

Protective sensation (Semmes-Weinstein 5.07/10g monofilament)

	Sites /3 (plantar hallux, plantar 1st MTPJ, plantar 5th MTPJ)
**Left foot**	
**Right foot**	

Note. Inability to detect the monofilament at ≥1 site(s) will indicate peripheral neuropathy. MTPJ = Metatarsophalangeal joint.

Arterial assessment

Pedal pulses

	Dorsalis pedis	Posterior tibial
**Left foot**		
**Right foot**		

Note. P = palpable, NP = not palpable.

Ankle-Brachial Pressure Index (ABPI) and Toe-Brachial Pressure Index (TBPI)

	Brachial systolic pressure (mmHg)	Ankle systolic pressure (mmHg)	Toe systolic pressure (mmHg)	ABPI	TBPI
**Left**					
**Right**					

Note. ABPI= ankle brachial index; TBPI= toe brachial index. ABPI≤0.9 and/or TBPI≤0.6 will indicate peripheral arterial disease and ABPI>1.3 will indicate arterial calcification.

DERMAL TEMPERATURE ASSESSMENT

Examiner 1 – Touch 1

**Date**	
**Participant #**	
**Charcot foot (Left/Right)**	
**Time post 15 minutes acclimatisation (minutes)**	
**Outside Temperature (°C)**	
**Room temperature (°C)**	

Site	Temperature (°C)
1	
2	
3	
4	
5	
6	
7	
8	
9	
10	

Examiner 1 – Touch 2

**Date**	
**Participant #**	
**Charcot foot (Left/Right)**	
**Time post 15 minutes acclimatisation (minutes)**	
**Outside Temperature (°C)**	
**Room temperature (°C)**	

Site	Temperature (°C)
1	
2	
3	
4	
5	
6	
7	
8	
9	
10	

Examiner 1 – Non-touch 1

**Date**	
**Participant #**	
**Charcot foot (Left/Right)**	
**Time post 15 minutes acclimatisation (minutes)**	
**Outside Temperature (°C)**	
**Room temperature (°C)**	

Site	Temperature (°C)
1	
2	
3	
4	
5	
6	
7	
8	
9	
10	

Examiner 1 – Non-touch 2

**Date**	
**Participant #**	
**Charcot foot (Left/Right)**	
**Time post 15 minutes acclimatisation (minutes)**	
**Outside Temperature (°C)**	
**Room temperature (°C)**	

Site	Temperature (°C)
1	
2	
3	
4	
5	
6	
7	
8	
9	
10	

Examiner 2 – Touch 1

Date	
Participant #	
Charcot foot (Left/Right)	
Time post 15 minutes acclimatisation (minutes)	
Outside Temperature (°C)	
Room temperature (°C)	
**Site**	**Temperature (°C)**
1	
2	
3	
4	
5	
6	
7	
8	
9	
10	

Examiner 2 – Touch 2

Date	
Participant #	
Charcot foot (Left/Right)	
Time post 15 minutes acclimatisation (minutes)	
Outside Temperature (°C)	
Room temperature (°C)	
**Site**	**Temperature (°C)**
1	
2	
3	
4	
5	
6	
7	
8	
9	
10	

Examiner 2 – Non-touch 1

Date	
Participant #	
Charcot foot (Left/Right)	
Time post 15 minutes acclimatisation (minutes)	
Outside Temperature (°C)	
Room temperature (°C)	
**Site**	**Temperature (°C)**
1	
2	
3	
4	
5	
6	
7	
8	
9	
10	

Examiner 2 – Non-touch 2

Date	
Participant #	
Charcot foot (Left/Right)	
Time post 15 minutes acclimatisation (minutes)	
Outside Temperature (°C)	
Room temperature (°C)	
**Site**	**Temperature (°C)**
1	
2	
3	
4	
5	
6	
7	
8	
9	
10	
